# Assessment of reporting sites for acute flaccid paralysis surveillance in Ethiopia: implications for planning of active case search visits

**DOI:** 10.11604/pamj.supp.2017.27.2.10731

**Published:** 2017-06-09

**Authors:** Eshetu Wassie, Ayesheshem Ademe, Kathleen Gallagher, Fiona Braka, Berhane Beyene, Abyot Bekele Woyessa, Daddi Jima

**Affiliations:** 1 World Health Organization, Ethiopia Country Office; 2 World Health Organization, Nigeria Country Office; 3 National Polio Laboratory, Ethiopian Public Health Institute; 4 Center for Public Health Emergency Management, Ethiopian Public Health Institute

**Keywords:** Acute flaccid paralysis, reporting sites, contribution, active surveillance visit, Ethiopia

## Abstract

**Introduction:**

The World Health Organization acute flaccid paralysis (AFP) surveillance standards recommend documentation of the role of each potentially reporting site for evidence -based planning and tailoring support for active surveillance visits. This study assessed the contribution of various sites as source and quality of AFP cases reported over a five -year period in Ethiopia.

**Methods:**

We conducted a retrospective analysis of AFP surveillance data collected from 2010-2014 in Ethiopia. Analyses were done using EPI-INFO version 7 for calculating frequencies and proportions, and testing possible associations between reporting sites and key dependent variables.

**Results:**

Of the 5,274 AFP cases reported, hospitals and health centers reported 4627 (88%) of the cases. Hospitals in Addis Ababa (53%) and health posts in Benishangul Gumuz (48%) regions have contributed majority of the cases reported. Only 3% of cases were reported by private clinics nationally. The stool adequacy rate for health posts (81%) was lower than the overall national rate of 88% .Cases from health posts are more likely to be reported after 14 days of onset of paralysis, and 62% less likely to be investigated within two days of notification(OR: 1.82, 95% CI OR : 1.41-2.36, p-value <0.0001). Greater proportion (2.4%) of cases reported from health posts were either compatible, VDPV or WPV compared to cases reported by health centers (1.14%) or hospitals (1.4%).

**Conclusion:**

Though majority of the cases were reported by health centers followed by hospitals ,our findings suggest that all potentially reporting sites should be exhaustively identified, prioritized and regularly supported for quality case detection, investigation and reporting.

## Introduction

In 2015 Ethiopia had an estimated population of 90 million living in nine Regional States and two City Administrations [1, 2].The pyramidal age structure of the population has remained predominately young with 44.9% under the age of 15 years [2, 3]. To reach the vast and predominantly rural population, Ethiopia has designed a unique flagship Health Extension Program (HEP), which delivers cost-effective basic health services to all Ethiopians, mainly women and children [4]. This innovative approach is defined by the core principle of community ownership that empowers communities to manage health problems specific to their communities, thus enabling them to determine their own health [5, 6]. To implement this program, about 15,618 health posts were constructed and are staffed by two health extension workers each, mainly female and recruited from the kebele (the lowest administrative structure). To enhance integrated service provision, health posts are linked with nearby health centers in the same administrative boundary [6]. According to the 2014 Service Provision Assessment Survey , a total of 3,292 health centers and 3,990 private clinics were functional during 2014 in addition to these health posts [7]. The survey also revealed that there were 202 functional hospitals; ongoing construction of an additional 123 hospitals was reported from seven regions [6].

The World Health Organization (WHO) recommends four inter-related strategies for global eradication of polio. These strategies include routine immunization, supplemental immunization activities (SIAs), surveillance, and targeted “mop-up” campaigns [8, 9]. Of these four strategies, acute flaccid paralysis (AFP) surveillance underpins the entire polio eradication program by monitoring progress of the implementation in other strategies. Without surveillance, it would be impossible to pinpoint where and how wild poliovirus(WPV) is circulating, or to verify when the virus has been eradicated in the world [8, 10]. Ethiopia started the polio eradication activities in 1996 following the Declaration on Polio Eradication in Africa in the same year and has been fully implementing the four strategies since then [11].

The first links in the surveillance chain are staff in all health facilities - from the smallest health post to the largest hospital. In areas with few formal health workers, some countries use community surveillance, where traditional healers, religious leaders or other community informants serve as a source of information on paralyzed children [10]. Apart from promptly reporting every AFP case, public health staff make regular visits to health facilities and other reporting sites to search for AFP cases which may have been overlooked or misdiagnosed [9, 10, 12]. For surveillance to detect all possible AFP cases, all these potential reporting sites should be exhaustively identified and adequately supported through regular visits. WHO surveillance standards for AFP recommend that the frequency of active case search visits should take into account, among other factors, the case detection and reporting experiences of the health facilities considered [11]. Limited WHO field staff along with a rapid expansion of health facilities in Ethiopia requires evidence-based planning and prioritization for sites to be regularly visited. There is a need to support surveillance site prioritization with systematically collected evidence. We conducted a study to assess the contribution of various sites as sources of AFP cases reported from 2010 to 2014 (a 5-year period) in Ethiopia. Various quality indicators of AFP surveillance were also analyzed by type of reporting site.

## Methods

We conducted a retrospective analysis of AFP surveillance data collected from 2010 to 2014. We extracted the data from the national AFP surveillance dataset that is maintained by the Expanded Programme on Immunization (EPI) Team of the WHO Ethiopia Country Office. We included only cases for which a reporting site was known in the dataset. Those cases with missing information on reporting site, date of notification, vaccination status (on both case-based investigation form and upon validation by field officers), stool condition and final classification status were excluded from our analysis. We included the following variables in our analysis: socio-demographic characteristics of cases (primarily age and place of residence), reporting site, dates of case notification and investigations, validation status, date of onset of paralysis, oral polio vaccine (OPV) vaccination status, dates of sample collection and condition of stool sample on submission to National Polio Laboratory, laboratory results and final classification of cases. We used the following operational definitions to describe the data:

**Reporting site:** the site where the case was initially notified, investigated and stool sample collected by a health worker.

**Good stool condition:** a case of AFP that fulfills the following criteria as evaluated by the National Polio Laboratory: two stool samples of at least 8 grams each collected 24 hours apart, transported and received with a temperature not exceeding 8°c. Additionally, the sample should be properly labeled and arrive within 72 hours of sample collection [13, 14].

**Late case:** an AFP case for which the second stool specimen is collected more than 14 days after of onset of paralysis.

**Discarded AFP case (Non-polio case):** 1) an AFP case where two adequate stool specimens are submitted for analysis but no poliovirus is isolated, or 2) an AFP case where the stool specimens are deemed inadequate but where there is no residual paralysis after 60 days of onset of symptoms [14].

**Compatible polio case:** 1) an AFP case that has inadequate stool specimens and has residual paralysis after 60 days of onset of paralysis, or 2) an AFP case that is lost to follow up or dies before detailed clinical examination is done after 60 days of symptom onset [14].

**Variable categorization:** the time between onset of paralysis and second stool collection dates were classified into ≤14 days and >14 days. Data on reporting site was classified into health post, health center, hospital, private health facility, and other sites such as holy water and traditional healer sites. OPV vaccination status was categorized into known (i.e., labeled with exact number of doses) or unknown upon initial investigation by the reporting sites. The stool condition upon submission was classified as good or bad as measured by the sample reception desk of the National Polio Laboratory. Lastly, final classification of reported cases was grouped into discarded, compatible, wild polio virus (WPV) or vaccine-derived polio virus (VDPV), and non-AFP.

**Data analysis:** analyses were done using Epi Info version 7 (US Centers for Disease Control and Prevention). Frequencies and proportions were calculated for the key variables and presented using tables, graphs and narrative. Odds ratio and chi-square for trend were calculated to determine possible associations between reporting sites and key variables such as onset of paralysis, notification and investigation, stool collection and condition, vaccination status, and final classification. The results were interpreted using 95% confidence intervals and p-value of 0.05.

**Ethical Clearance:** there were no human subjects involved in this study and there was no need to obtain written permission.

## Results

**Figure 1:**
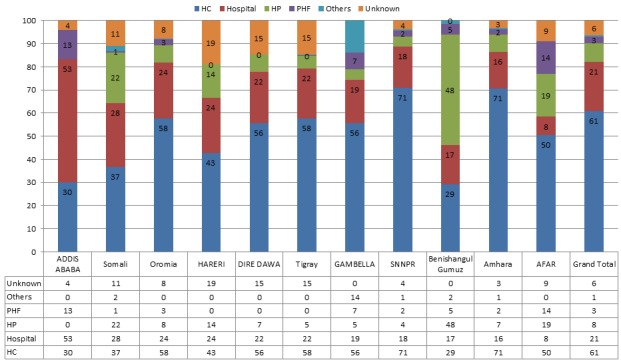
Distribution (%) of AFP cases by type of reporting sites and region, Ethiopia, 2010-2014

Between 2010 -2014, 5,274 AFP cases were reported; the majority (65%) were reported by health centers. Hospitals and health centers jointly reported 88% of the cases. The contributions of health posts and private health facilities in AFP case reporting were low (9% and 3%, respectively) (Table 1). The overall stool adequacy rate for cases reported from all types of reporting sites during 2010-2014 was 88%. The rate was relatively lower for cases reported from health posts (81%) and hospitals (87%) compared to other sites. Cases reported by health posts were more likely to be investigated and reported after 14 days of paralysis than other sites (OR: 1.82, 95% CI: 1.41-2.36, p-value: <0.00001) (Table 2). Health centers reported more than two-thirds of the AFP cases in Amhara (71%) and the Southern Nations, Nationalities, and Peoples' Region (SNNPR) (71%). Hospitals reported more than half of the cases in Addis Ababa (53%). Health posts reported about half (48%) of the cases in Benishangul-Gumuz and one-fifth of the cases in Somali (22%) and Afar (19%). Private health facilities reported about 13% of the cases in Addis Ababa and 14% of the cases in Afar (Figure 1).

**Table 1:** Distribution of AFP cases by reporting sites and year, Ethiopia, 2010-2014

	Reported Cases by Year					
Reporting site	2010 n (%)	2011 n (%)	2012 n (%)	2013 n (%)	2014 n (%)	Total n(%)
Health Center	647(61)	553(62)	709(66)	758(68.5)	755(67)	3422(65)
Hospital	251(24)	227(26)	256(24)	220(20)	251(22)	1205(23)
Health Post	101(10)	83(9.3)	77(7)	89(8)	103(9)	453(9)
Private health facilities	58(5.5)	23(2.6)	27(2)	34(3)	16(1)	158(3)
Others	4(0.4)	3(0.3)	12(1)	6(0.5)	11(1)	29(1)
**Total**	**1061(100)**	**889(100)**	**1081(100)**	**1107(100)**	**1136(100)**	**5274(100)**

**Table 2:** Stool adequacy rate (%) by reporting site, Ethiopia, 2010-2014

	Adequate samples		No. investigated after 14 days of paralysis onset		Odds Ratio (95% CI); p-value
Reporting site	N	%	n	%	
Health post	367	81	86	19	OR: 1.82 ( all other sites) (95% CI: 1.41-2.36) p-value: <0.00001
Hospital	1048	87	157	13	
Health center	3046	89	376	11	
Private health facility	145	92	13	8	
Others	33	92	3	8	
**Total**	**4641**	**88**	**633**	**12**	

Twenty-four percent of the reported 5,274 AFP cases were with unknown OPV vaccination status. AFP cases reported by hospitals were more likely to have unknown vaccination status than cases reported from health posts (OR: 1.92, 95% CI: 1.45-2.55, p-value: <0.001), while cases reported from health centers were more likely to have unknown vaccination status than those reported from health posts (OR: 1.71, 95% CI: 1.3-2.25, p-value: <0.001). About 13% of the reported cases had no history of OPV vaccination ("zero dose") (Table 3). Almost all (97%) of the reported AFP cases were investigated within 2 days of notification. Timeliness of investigation was lower in cases reported from health posts (93%) compared to those reported from other sites (97%). The odds of being investigated within two days of notification for cases reported by health posts were 62% lower than by other sites (OR: 0.38; 95%CI: 0.26-0.57 p-value <0.0001) (Table 4).

**Table 3:** Distribution of unknown opv vaccination status from case investigation form of afp cases by reporting site, Ethiopia, 2010-2014

	Unknown		Known		Odds ratio (95% CI)
Reporting Site	N	%	n	%	
Health posts	71	16	382	84	1.00
Private health facilities	36	23	122	77	1.59 (1.01-2.49)
Health Center	825	24	2597	76	1.71(1.31-2.23)
Others	9	25	27	75	1.79(0.8-3.97)
Hospital	317	26	888	74	1.92(1.45-2.55)
**Total**	**1258**	**24**	**4016**	**76**	X^2^ = 15.48658 p-value: 0.00008

**Table 4:** Comparison of Timeliness of Investigation of Notified Cases by Reporting Site, Ethiopia, 2010-2014

	Investigated within 2 days	Investigated after 2 days	Total cases investigated	Odds Ratio (95% CI), p-value
Reporting Site	n(%)	n(%)	N(%)	
Health posts	420(93)	33(7)	453(100)	1.0 OR: 0.38 (Health post vs other sites) (95%CI: 0.26-0.57) p-value <0.0001
Health Center	3327(97)	95(3)	3422(100)	
Hospital	1165(97)	40(3)	1205(100)	
Private health facilities	153(97)	5(3)	158(100)	
Others	35(97)	1(3)	36(100)	
**Total**	**5100(97)**	**174(3)**	**5274(100)**	

**Figure 2:**
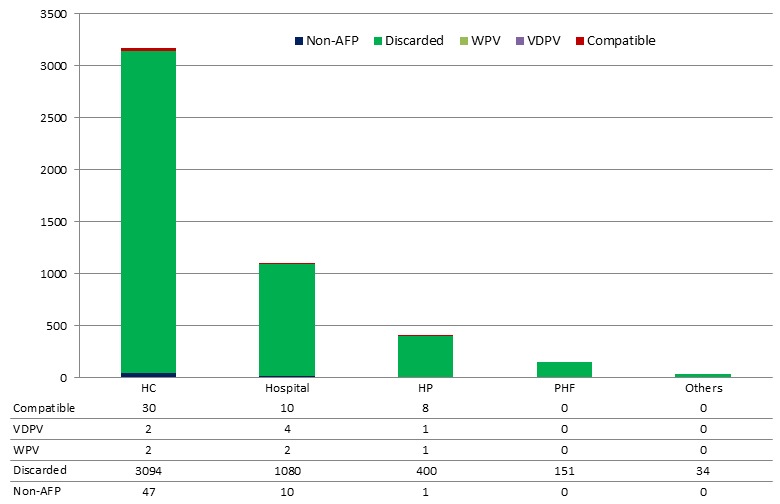
Final classification of AFP cases by reporting sites, Ethiopia, 2010-2014

Of the 5,143 cases reported with the condition of stool recorded upon submission to the National Polio Laboratory, 86% were in good condition. Stool condition was below the target of 90% for all types of health facilities. Cases reported from private health facilities had lowest good stool condition (84%) compared to the other reporting sites. Stool samples collected from hospitals were more likely to be in good condition than those reported from health centers and this association was statistically significant (OR: 1.25, 95% CI: 1.02-1.53, p-value: 0.03) (Table 5). Of the 4,877 suspected AFP cases with known classification status, 4,759 (97.6%) were discarded as non-polio AFP cases. A total of 12 (0.2%) of these suspected cases were confirmed to be wild or vaccine derived poliovirus cases. One percent of the cases were classified as compatible polio cases. About 2.4%, 1.4% and 1.14% of cases reported from health posts, hospitals and health centers were either compatible, VDPV or WPV, respectively (Figure 2).

**Table 5:** Stool condition by reporting site, Ethiopia, 2010-2014

	Good		Bad		Odds Ratio(95% CI))
Reporting Site	n	%	N	%	
Hospital	1038	88	139	12	1.00
Health Center	2851	86	478	14	0.799(0.65-0.98)
Health Post	378	85	66	15	0.761(0.56-1.1)
Private health facilities	132	84	25	16	0.707(0.44-1.12)
Others	34	94	2	6	2.276(0.57-19.8)
**Total**	**4433**	**86**	**710**	**14**	

Odds ratio for hospital versus health center: 1.25 (95% CI: 1.02-1.53, p-value: 0.03)

## Discussion

Our study found that a greater proportion of AFP cases were reported by health centers (65%) and hospitals (23%) compared to other sites. Only 9% of cases were reported by health posts. Though private health facilities reported a much larger proportion of cases in Addis Ababa (13%) and Afar (14%) compared to other regions, their contribution in AFP notification and investigation was low as they contributed only 3% of the reported cases. When case reporting by type of health facility was further analyzed for each region, most of the cases were reported by health centers. However, majority of cases in Addis Ababa (53%) and Benishangul-Gumuz (48%) were reported by hospitals and health posts, respectively.

The contribution of health posts in AFP case notification was also much more significant in relatively underdeveloped and mostly pastoralist regions of the country, where health posts contributed about one-fifth of the AFP cases reported over the 5- year study period. This is a justification for need of scaling up and further strengthening of community-based surveillance in these regions. Low detection and notification of cases by private health facilities in Ethiopia might partly be due to unfamiliarity with the AFP case definition, lack of training or inadequate supervision by surveillance staff. Similar findings have been reported from other countries. A study conducted in Egypt showed that lack of knowledge on notification and investigation procedures played a role in low contribution of private facilities in AFP surveillance [15]. Though widespread in distribution throughout Ethiopia, contributions of holy water and other traditional sites were negligible (1%).This may be due to lack of proper documentation about notifying sites on the AFP case investigation form.

We also found that cases reported by health posts were more likely to be notified and investigated after 14 days of onset of paralysis. Case investigations are also delayed after being notified by health posts. Cases reported from health posts are less likely to be investigated after two days of notification. All these are against the standard recommendations for a sensitive AFP surveillance system [16]. Considering the increased accessibility of health posts to the community when compared with other types of health care facilities, this is an unexpected finding. The reasons for delay in notification and investigation at health post level might be due to irregular active case search visits by health extension workers, weak linkage with the community, delays in notification to health centers and woredas upon detecting suspected cases, and delay in case verification and sample collection and shipment by health workers at health centers after getting the call from health posts. A study in Luanda, Angola revealed similar findings that AFP cases pass through different levels of the system before they come to the attention of a more qualified health worker. This was considered as one cause of delay in actual case investigation and notification [17].

Another striking finding is that much higher proportion (2.4%) of cases reported from health posts were compatible polio, VPDV and WPV cases than those reported from other sites. The National Polio Expert Committee classifies cases as polio compatible when there is no adequate clinical, immunization and laboratory information to rule out with certainty the possibility of poliomyelitis [13]. These findings are contrary to the existing belief that increasing access to health services to communities can shorten the time lag between disease onset and care seeking for timely and appropriate interventions [18, 19]. Lack of follow up or inadequate investigation of late cases by surveillance officers also increases the likelihood of compatible polio cases [10].

A positive finding for health posts is that cases reported by health posts were more likely to have a properly recorded OPV immunization status when compared to those cases notified by health centers and hospitals. This might be due the fact that the vaccination status of cases can be retrieved from their cards or immunization records kept at health posts, much nearer to the residence of cases. Moreover, most health extension workers, the primary vaccinators in the community, are more familiar with the children vaccination status. We also examined if there are obvious differences in quality of stool sample collection, transportation and condition on submission by type of reporting sites. Cases reported from health centers were more likely to be in bad condition than those reported from hospitals. The possible reason might be due to lack of direct public transport connections of these health centers with Addis Ababa, where the central polio laboratory is located, and the relatively longer distances to be travelled by sample transporters coming from health centers.

The primary limitation of our study is that the case investigation form, the primary data collection tool, was not designed to document each and every detail of case notifications. It lacks sections to clearly record contributions of traditional sites and other community level system in case notification. Thus, it is highly likely that the role of community in case notification is underestimated. The other limitation of the study is that the amount of active case search varies significantly by type of health facility and this could lead to varying degrees of underreporting. However, this limitation does not change the impression that each site providing traditional or conventional health care has a role in AFP surveillance.

Our findings suggest that regardless of the region, all sites have a potential to report AFP cases of varied quality and quantity. However, in situations where logistics, personnel and time are insufficient, sites with a good track record of detecting and reporting, and those silent for relatively long time should be prioritized and visited without undermining the contribution of other sites.

## Conclusion

We conclude that, almost all cases are reported by health centers and hospitals in SNNPR, Amhara, Tigray and Oromia, the bigger regions of the country. Private health facilities in Addis Ababa and health posts in Benishangul-Gumuz, Somali and Afar have made significant contributions in AFP case notification and reporting. Cases reported by health posts are relatively lower quality. We recommend that all potentially reporting sites should be exhaustively identified, prioritized and feasible strategies designed to be regularly supported for quality case detection, investigation and reporting. Private health facilities in major towns of each region should also be targeted for surveillance strengthening. Furthermore, a review of the case investigation tools to determine the contribution of traditional sites in case reporting would increase the evidence for further strengthening of the surveillance system at community level.

### What is known about this topic

- Health facilities are important sources of acute flaccid paralysis cases. Thus, epidemiologists should prioritize and make regular visits.

### What this study adds

- Assessing the contribution of each health facility is critical for evidence based decision-making on subsequent planning of active case search visits;
- This issue is getting greatest attention in settings where health facilities are massively expanding while the number of health workers for visiting increasing numbers of health facilities for surveillance is still limited.

## Competing interests

The authors declare no competing interests. The views expressed in the perspective articles are those of the authors alone and do not necessarily represent the views, decisions or policies of the institutions with which they are affiliated and the position of World Health Organization.
